# Structure and Evolution of *Streptomyces* Interaction Networks in Soil and In Silico

**DOI:** 10.1371/journal.pbio.1001184

**Published:** 2011-10-25

**Authors:** Kalin Vetsigian, Rishi Jajoo, Roy Kishony

**Affiliations:** Department of Systems Biology, Harvard Medical School, Boston, Massachusetts, United States of America; University of California Davis, United States of America

## Abstract

Soil grains harbor an astonishing diversity of *Streptomyces* strains producing diverse secondary metabolites. However, it is not understood how this genotypic and chemical diversity is ecologically maintained. While secondary metabolites are known to mediate signaling and warfare among strains, no systematic measurement of the resulting interaction networks has been available. We developed a high-throughput platform to measure all pairwise interactions among 64 *Streptomyces* strains isolated from several individual grains of soil. We acquired more than 10,000 time-lapse movies of colony development of each isolate on media containing compounds produced by each of the other isolates. We observed a rich set of such sender-receiver interactions, including inhibition and promotion of growth and aerial mycelium formation. The probability that two random isolates interact is balanced; it is neither close to zero nor one. The interactions are not random: the distribution of the number of interactions per sender is bimodal and there is enrichment for reciprocity—if strain A inhibits or promotes B, it is likely that B also inhibits or promotes A. Such reciprocity is further enriched in strains derived from the same soil grain, suggesting that it may be a property of coexisting communities. Interactions appear to evolve rapidly: isolates with identical 16S rRNA sequences can have very different interaction patterns. A simple eco-evolutionary model of bacteria interacting through antibiotic production shows how fast evolution of production and resistance can lead to the observed statistical properties of the network. In the model, communities are evolutionarily unstable—they are constantly being invaded by strains with new sets of interactions. This combination of experimental and theoretical observations suggests that diverse *Streptomyces* communities do not represent a stable ecological state but an intrinsically dynamic eco-evolutionary phenomenon.

## Author Summary

Soil harbors a diverse spectrum of bacteria that secrete small molecules such as antibiotics. *Streptomyces* bacteria, considered the most prolific producers, have been mined for decades for novel products with therapeutic applications, yet little is known about the properties of the interaction networks these compounds mediate. These networks can hold clues about how the diversity of small molecules and of *Streptomyces* strains with different production and resistance capabilities is maintained and promoted. To explore the network properties, we developed a high-throughput platform for measuring pairwise phenotypic interactions mediated by secreted metabolites, and used it to measure the interaction network among 64 random *Streptomyces* isolates from several grains of soil. We found many strong but specific interactions that are on average determined more by metabolite production than by metabolite sensitivity. We found reciprocity between strains, whereby if one strain inhibits or promotes the growth of a second strain, it's likely that the second strain affects the first strain in a similar manner. These interactions are not correlated with phylogeny, as very closely related strains exhibit different interaction patterns. We could explain these findings with a mathematical model requiring interplay between ecological dynamics and evolution of antibiotic production and resistance, suggesting that the bacterial and small molecule diversity of these communities is maintained by constant evolutionary turnover of interaction phenotypes.

## Introduction

Sampling DNA from diverse ecosystems has revealed a breathtaking diversity of microbial life [1],[2], especially in soil [3]–[5]. But we have barely begun to explore, both experimentally and theoretically, how these complex communities coexist and function. We know that microbes can interact via secretion of a wide array of small molecules, most notably antibiotic compounds. But how prevalent, diverse, and specific are such interactions? How is the incredible diversity of microbes and their natural products maintained and promoted by complex and spatially structured networks of interactions?

**Figure 1:**
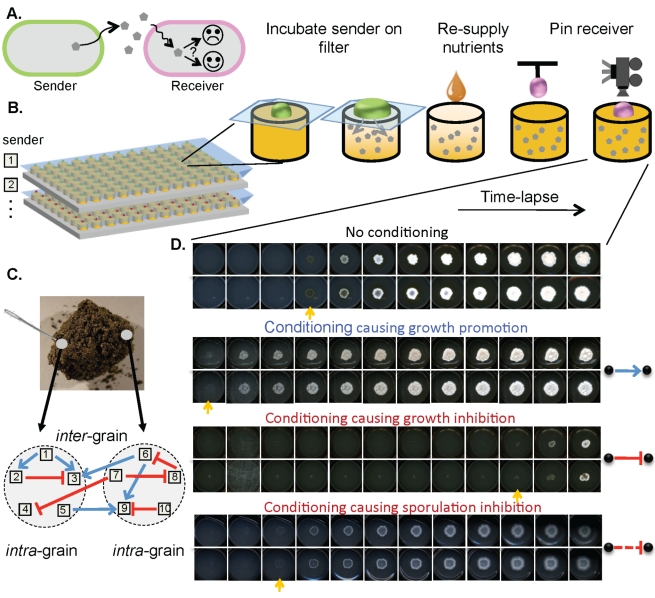
Platform for high-throughput measurement of pairwise interactions. (A) A directional pairwise interaction measures how the products of a sender strain affect the morphological colony development of a receiver. (B) A fine-pored filter is placed on an agar surface, a lawn of the sender is grown on top, and the filter is removed, leaving behind sterile agar that has been altered (*conditioned*) by the sender strain. The conditioned agar is then resupplied with concentrated liquid nutrients to compensate for the nutrients consumed by the donor. A receiver strain is pinned (point-inoculated) onto agar and imaged frequently, which allows us to see the developmental phenotype of the receiver. (C) Grains were sampled with a needle tip from soil cores, and *Streptomyces* strains isolated. (D) By comparing colony growth of the receiver strain on conditioned and non-conditioned agar, we can identify interactions between strains. Shown is the colony development of a receiver in two replicas on non-conditioned and three conditioned media (on a subset of time points). The colony appearance time is marked by orange arrow. We observed partial or complete inhibitions of growth, faster colony appearance, inhibition of aerial mycelium formation, as well as (not shown) changes in colony morphology and sporulation enhancements. We have developed a high-throughput implementation of this technique using 96-well agar plates, robotic inoculation of the sender, nutrient resupply and pinning, an array of 30 modified optical scanners, and automated data acquisition and image analysis.

To tackle these questions, we isolated bacterial strains from individual grains of soil and systematically measured all pair-wise interactions among them. We measured compound-mediated interactions, where a “sender” strain affects a “receiver” strain by secreting metabolites, antibiotics, or other compounds (Figure 1AB). We focused on bacteria from the genus *Streptomyces*, which are the most prolific producers of small molecules, are abundant in soil [6], and exhibit diverse production and resistance capabilities [6]–[8] that are modular and prone to Horizontal Gene Transfer (HGT) [9]. Sixty-four *Streptomyces* from four individual grains of soil were isolated (Figure 1C), phenotyped for all possible pair-wise interactions, and genotyped for 16S rRNA. We explored the statistical properties of the resulting network and juxtaposed them with those emerging from a simple ecological model of bacteria evolving production of and resistance to antibiotics [10],[11].

We developed a high-throughput platform for measuring directional pairwise interactions by observing how the products of one bacterial strain affect the colony growth of another. A fine-pored filter is placed on a nutrient agar surface, a lawn of the sender strain is grown on top, and the filter is removed—leaving behind sterile agar that has been altered or *conditioned* by the sender strain. The conditioned agar is then resupplied with concentrated liquid nutrients to compensate for the nutrients consumed by the donor. A receiver strain is point-inoculated onto the sterile conditioned agar and time-lapse movie of the growing colony is taken. A high-throughput implementation of this assay allowed us to acquire 11,500 movies along 15 d at 4 h time resolution, covering all pairwise interactions within our collection of 64 strains in duplicate (Figure 1D, Materials and Methods). By comparing colony growth of the receiver strains on conditioned and non-conditioned agar, we identify interactions between strains. We quantified the first time point in which each colony becomes visible on the images (appearance time) to identify growth inhibitory and growth promotion interactions. In addition, we visually scored instances of inhibition of aerial mycelium formation (and subsequent sporulation).

## Results

### The Interaction Network Is Balanced, Diverse, and Sender-Determined

**Figure 2:**
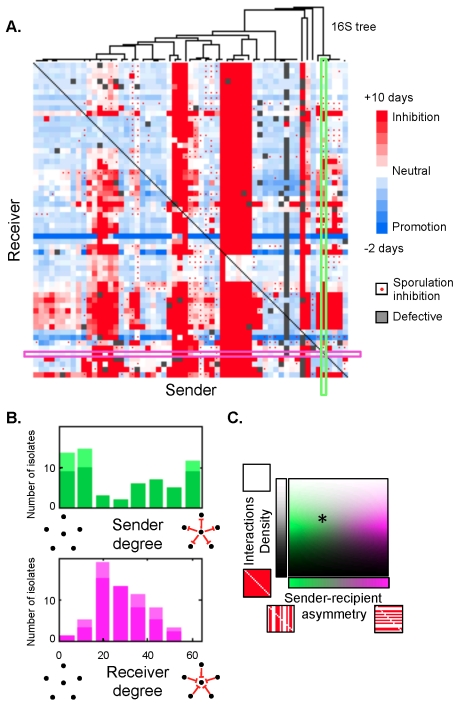
Interactions are balanced and sender-determined. (A) Interaction matrix sorted by 16S relatedness. The sender isolate is on *x*-axis, receiver isolate on the *y*-axis. The color of each entry is determined by the appearance time of a receiver colony on conditioned media relative to non-conditioned media: later appearance (red), earlier appearance (blue), no effect (white), and missing data (gray). Color saturation increases with the magnitude of the effect, with full saturation reached for 10 d delay (red) and 2 d speedup (blue). Red dots indicate inhibition of aerial mycelium formation. Five isolates having sender or receiver information missing are not shown. (B) Sender and receiver degree distributions for inhibitory interactions (appearance time and sporulaton inhibitions combined). The light-colored portion of the bars displays the contribution from “clonal” isolates (identical interactions and 16S). Here, negative interactions are defined as delays of more than 1 d in colony appearance time or as inhibitions of aerial mycelium formation. (C) Shown is the position of the observed matrix (star) in the space formed by the fraction of interactions and the sender-receiver asymmetry. The color expresses the sender-receiver asymmetry (from green for sender-determined to magenta for receiver-determined) and the lightness expresses the fraction of inhibitory interactions (from white for no interactions to black for all strains interacting with all others).

We found a rich and complex interaction matrix among our collection of strains with multiple cases of growth inhibition, growth enhancement, and inhibition of aerial mycelium (Figure 2A; and see also all the 11,500 time lapse movies underlying this matrix in Figure S8). This matrix showed two immediately apparent special properties: it is “balanced” and it is “sender determined,” as we now explain.

The first noteworthy property of the matrix is that the frequency of interactions (a.k.a. connectance) is balanced: the probability that two random isolates interact is neither close to zero nor close to one. While there are many strong interactions, the matrix is far from the limit in which interactions are non-specific because everyone interacts with everyone else: of the 64 isolates, there are at least 42 different interaction profiles. We found 45% of growth or aerial mycelium inhibitory interactions (25% complete inhibitions of growth) and 19% growth promotion (see Materials and Methods for additional details). The frequency of inhibitory interactions is significantly higher (and more balanced) than previous estimates based on zones of inhibition [12]. The balanced frequency of interactions makes this network more highly connected than most known ecological networks (only for a few food webs the density approaches 30%) [13]–[16].

The second striking property of the measured matrix is that it is very different along the sender and receiver axes, with characteristic stripes of inhibition and non-inhibition running along the receiver (vertical) axis. This asymmetry is surprising because the existence of an inhibitory interaction is, in general, influenced by both the sender, which needs to produce a toxic compound, and the receiver, which needs to be sensitive to the compound produced. In the extremes, a matrix that is determined purely by the properties of the sender would exhibit perfect stripes in the vertical direction, while a receiver-determined matrix would exhibit stripes along the horizontal (sender) axis. Thus, the network we observed is more sender-determined. This sender-receiver asymmetry can be quantified by comparing the distribution of the fraction of isolates that each isolate inhibits (*sender degree*) with the distribution for the fraction of isolates that inhibit each isolate (*receiver degree*) (see Figure 2B). The sender degree is broad and peaks near its extreme values, while the receiver degree is narrower and unimodal. The difference of the variances of the receiver and sender distributions is a measure of the *sender-receiver asymmetry* (Q = −0.37; Figure 2C). The negative value of this quantity means that information gathered about a sender from a few interactions let us predict far better the rest of its interactions than the corresponding information about a receiver. The sender-receiver asymmetry is pronounced but not extreme, indicating the importance of resistance to antibiotics that strains do not themselves produce. The bimodality of the sender degree distribution makes this network very different from networks with nodes randomly connected with a fixed probability (Erdös–Renyi random graphs), scale-free (social) networks [13], and food webs with exponential-tailed distributions [17].

It is unclear how coexistence between strains that inhibit almost everyone and strains that inhibit almost no one is maintained. One possibility is the presence of an ecological tradeoff between ability to inhibit and ability to resist, which would imply a positive correlation between the sender and receiver degrees. But no such correlation exists; on the contrary, isolates that inhibit most are also among the most resistant (Figure S1A, p<10^−4^). There is also no correlation between growth rate on non-conditioned media and the sender or receiver degree (Figure S1B).

**Figure 3:**
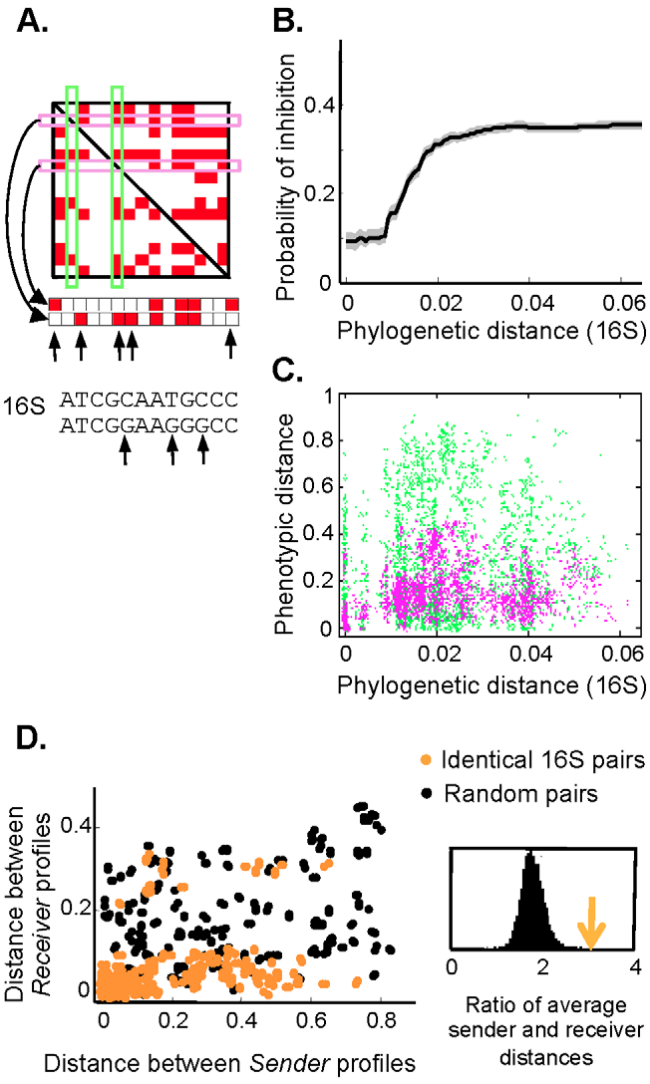
Sender profiles diverge faster than receiver profiles. (A) Distance between a pair of sender/receiver profiles is defined as the fraction of differing positions, and phylogenetic distance is proportional to the number of base pair differences in 16S rRNA. (B) Closely related isolates are less likely to inhibit each other. Plotted is the probability of inhibition for all pairs of isolates closer than a given 16S rRNA distance. (C) Number of 16S nucleotide differences (*x*-axis) versus number of sender (green) and receiver (magenta) profile differences (*y*-axis) for all isolate pairs. (D) The distance between sender profiles is plotted against the distance between receiver profiles for pairs with identical 16S rRNA (orange circles), and for pairs with identical randomized 16S (black circles). The randomization was done by permuting the assignments of the 16S sequences to isolates. There is an apparent tendency for sender profiles to diverge disproportionately more than receiver profiles for closely related strains. This is quantified in the inset, which displays the mean ratio of distances between sender and receiver profiles for pairs with identical 16S (orange arrow) and the corresponding distribution obtained by randomly permuting the assignments of 16S sequences to isolates (black histogram); *p*<10^−4^.

We decided to look for hints about the maintenance of a diverse sender-determined network in the network evolution. We sequenced the 16S rRNA of all isolates and found that closely related isolates are less likely to inhibit each other (Figure 3B), but there is a poor overall correlation between phenotypic and phylogenetic distances (Figure 3A,C). Even isolates with identical 16S rRNA sequences can have very different interaction profiles. This lack of strong correlation between phylogeny and inhibition profiles is consistent with previous work [18]. To further exploit this phylogenetic signal, we compared the phenotypic divergence of sender and receiver profiles for isolates with the same 16S, and contrasted it with the null expectation of isolates with different 16S (Figure 3D). Interestingly, the sender profiles diverge disproportionately more than the receiver profiles for closely related strains even after controlling for the overall sender-dominated nature of the matrix (P = 2⋅10^−4^). So it seems that the *Streptomyces* community is in a state in which frequent evolutionary changes in production (mediated for example by transfer of plasmids carrying antibiotic production genes) cause dramatic changes to ecological interactions. The coupling between ecology and evolution is therefore important for understanding the network properties.

### Model

Is the balanced frequency of interactions and sender-determined nature accidental or a natural outcome of the ecological and evolutionary dynamics of interacting *Streptomyces* communities? Can we account for the large changes in interaction patterns over short evolutionary distances?

**Figure 4:**
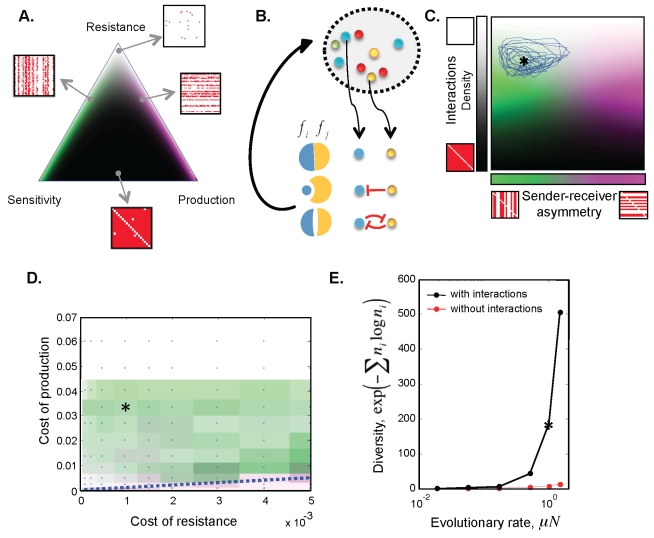
Balanced and moderately sender-determined matrices emerge naturally from an evolutionary model of antibiotic interactions. (A) Matrix statistics for in silico strains randomly assigned production, resistance, or sensitivity to 40 antibiotics. A point inside the triangle specifies the probabilities of producing, being resistant to, or being sensitive to antibiotics. The triangle vertices correspond to the cases where every community member is sensitive to, resistant to, or producer of all antibiotics. Probabilities are proportional to the distance from the opposing edge, and sum to 1. The color represents the fraction of interactions and the sender-receiver asymmetry in the color scheme used in (2C). (B) Schematic of the ecological dynamics. The fitness of a colony (e.g. number of spores it produces) is determined by the pairwise interactions with its neighbors. In the ecological dynamics we average over all possible combinations of neighbors. (C) An evolutionary trajectory, after statistical steady state has been reached, projected onto the plane of interaction density (*y*-axis) versus sender-receiver asymmetry (*x*-axis). The trajectory fluctuates around a mean value (denoted by *). (D) This mean value is calculated on a grid of costs of production and resistance (blue dots; patch color represents the mean interaction density and sender-receiver asymmetry, as defined in C). In a large area of parameter space, when production cost is larger than resistant cost (above the blue dotted line), the interaction matrix is balanced and sender determined (green shades). Very low resistant cost would lead to low density of interactions (not shown). (E) Functional diversity requires both ecological interactions and evolution. Functional diversity is shown as a function of evolutionary rate for simulations with species interactions (black circles) and with effect of interactions on the dynamics disabled (red dots). Two organisms are considered functionally distinct if they interact differently (as senders or receivers) with an existing organism. Diversity is expressed as the exponential of the Shannon-Wiener Index, and the population evolutionary rate is µN (plotted for fixed population size *N* = 10^6^ and varied mutation rate μ). The stars in (C), (D), and (E) correspond to the same simulation, the dynamical properties of which are shown in Figure S4.

We consider a simple in silico model of communities of strains producing and resisting a set of antibiotics. A strain inhibits another if it produces at least one antibiotic to which the other is sensitive. Communities of strains with randomly assigned production of and resistance to antibiotics exhibit a diverse set of qualitatively different interaction matrices, depending on the frequency of production and resistance (Figure 4A). With many antibiotics, matrices similar to the observed—balanced frequency of interactions and moderately sender-determined—occupy a small region of the parameter space, and require low frequency of production and high frequency of resistance. This raises the question of whether introducing evolution into the model can inherently direct it into the regime of balanced and sender-determined interactions.

We imposed simple non-spatial ecological dynamics that implicitly incorporates the importance of spatial relations between bacteria over short time scales (the antibiotics stay near their producers). The fitness of each strain depends on the weighted sum of its interactions with all other strains, incorporating the following contributions: (i) a negative effect of being inhibited by others, (ii) an advantage of inhibiting others, and (iii) a reduced ability to inhibit if being inhibited (*protection by inhibition*). A cost for production or resistance of any antibiotic is also added. The resulting mathematical structure is that of a discrete time Lotka-Volterra model with coefficients derived from the pairwise interaction matrix (Figure 4B and Materials and Methods).

For simplicity, the model ignores that antibiotics might also function as a “common good” reducing competition from non-resistant *Streptomyces* and non-*Streptomyces* strains. In addition, it ignores the possibility of resistant neighbors extending their protection to non-resistant strains [19]. We also ignore positive interactions and primary metabolism differences (utilization of different resources and cross-feeding), which are potentially important. Some of these effects can be incorporated by adding terms with higher order interactions (for several model extensions, see Materials and Methods and Figure S7).

To capture the long-term effects of the interplay between ecology and evolution on the statistical properties of interactions, we added mutations to the above model. Mutations allow acquisition or loss of production and resistance to any of the antibiotics. Turnover of production and resistance capabilities is indeed expected to be important for *Streptomyces*, as evidenced by the modular nature of antibiotic production and the vectors through which it spreads.

The simulation starts from a single strain that is sensitive to all antibiotics and follows the dynamics until a statistical steady state is reached (Figure 4C). We systematically explored the behavior of the model for a range of costs of production and resistance (Figure 4D). The results show a maximum cost of production above which no antibiotics are produced (Figure 4D, white area). Strikingly, below this threshold we see balanced sender-determined matrices (Figure 4D, green shades), as long as the production costs are higher than the resistance costs (above the dashed blue line). There is an inherent feedback that keeps the frequency of interactions from becoming too low or too high: an increase of interaction frequency selects for an increase in resistance levels, which then leads to a decrease of the interaction frequency. This qualitative picture holds provided that the level of protection by inhibition is below a certain threshold (Figures S2, S3A). On the other hand, if inhibition is an effective defense, then when resistance cost is high the system collapses into a state in which most strains inhibit each other (Figures S2, S3A). While different outcomes are possible in the model, we observe balanced and sender-determined matrices over a large region of the parameter space (Figure S3).

We also explored the relation between interaction and phylogeny in the simulations. In agreement with our experimental observation, in the balanced and sender-determined region, we find that in the resulting interaction matrices (Figure S4A) strains are more likely to interact when they are phylogenetically distant (Figure S4B), and there is a weak overall correlation between phylogenetic and phenotypic distance (Figure S4C).

Community diversity requires both ecology and evolution. The functional diversity of the system increases sharply with both the evolutionary rate and the population size, and turning off the ecological interactions or reducing the mutation rate leads to a loss of diversity (Figure 4E and Figure S5). The community steady state is characterized by a continuous turnover of different interaction phenotypes (Figure S4D), indicating its evolutionary instability.

### Reciprocity

**Figure 5:**
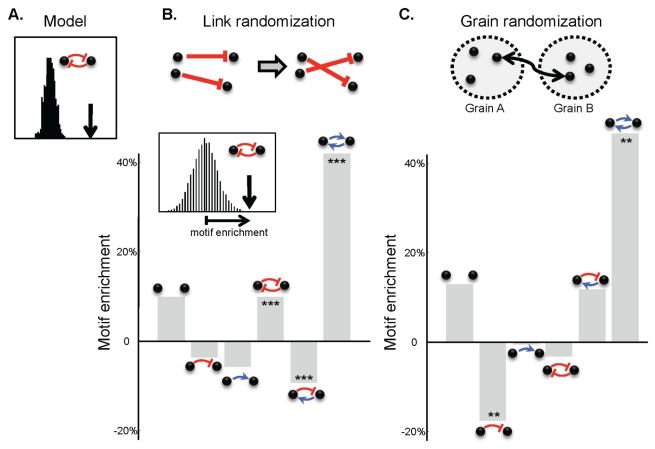
Reciprocity is enriched for all isolates and within grains. (A) The frequency of mutual inhibitions within a simulated network (arrow) is compared with that for random networks preserving the sender and receiver degrees of each node (histogram). (B) For the measured network of interactions, we compare the frequency of the six possible pairwise motifs of positive and negative interactions with those for suitably randomized networks. We constructed random networks with the same receiver and sender degrees for each isolate. We compared the motif frequency with that for the distribution for randomized networks (Inset). Bars show the relative deviation of actual motif frequencies from the mean for randomized networks, and stars indicate the significance. (C) Intra-grain motif frequencies reveal spatial structuring. We permuted the assignment of isolates into grains, and calculated the motif frequencies only for pairs of isolates from a same grain. For both panels, positive interactions are defined as at least 12 h earlier colony appearance, and negative interactions are as in Figure 2B. In (A) and (B) we included only isolates with distinct sender or receiver profiles.

To investigate statistical properties beyond those captured by the degree distributions, we followed an established procedure for identifying interaction motifs—local patterns of interactions that are more frequent than expected by chance [20]. We discovered that the simulated interaction networks are strongly enriched for mutual inhibition when compared with random networks with the same sender and receiver degrees for each isolate (Figure 5A). This is not surprising since mutual inhibition is an important mechanism for ecological balance. We, therefore, looked for reciprocity in the experimental data. In the experimental data, unlike the model, there is an extra complexity due to positive interactions (growth promotions are not included in the model). With both positive and negative interactions there are six two-isolate motifs (Figure 5B,C). We compared the six motif frequencies with those for random matrices that have the same sender and receiver degrees for each isolate, and which preserve the corresponding degrees for the growth promotion interactions. Since an obvious source of reciprocity structure is the presence of identical isolates, we excluded strains with identical 16S and interaction profiles from this analysis. As we observe in the model, the analysis of the experimental data revealed statistically significant enrichment for reciprocal interactions—there are more mutual inhibitory interactions and mutual growth promotions than expected and fewer asymmetric relationships (Figure 5B).

If reciprocity of interactions among pairs of strains is a property of coexisting communities, we may expect that it will be more enriched in strains coming from the same soil grain than for strains isolated from different grains. We found that while the frequency and strength of positive and negative interactions does not differ within and between grains, interactions of pairs of strains within grains do indeed tend to be more reciprocal than interaction of strains from different grains (Figure 5C). This result is significant only if we include the inhibitions of aerial mycelium. The motif distributions are also sensitive to the choice of thresholds for defining interactions. A threshold independent analysis of the continuous data shows again enrichment for reciprocity (Figure S6, *p* = 0.001). The apparent enrichment for reciprocity remains if we control for a tendency to have isolates with more similar 16S within a grain. A larger dataset will be required to distinguish between different underlying causes for the patterns of interactions within and between soil grains.

## Discussion

We find that *Streptomyces* isolates from soil grains exhibit diverse and rich interaction patterns. The interaction matrix they form has a balanced frequency of interactions—the probability that two random strains interact is neither close to zero nor to one. The sender-degree distribution is broad and bimodal—isolates tend to inhibit almost everyone or almost no one, which makes the interactions statistically controlled more by the properties of the sender than the receiver. This sender-receiver asymmetry, while pronounced, is not extreme, indicating the importance of resistance to compounds produced by others. These properties make this network very different from other ecological networks, which have monotonic degree distributions, and typically exhibit much lower interaction frequency. Finally, the community is enriched in reciprocal interactions—interaction pairs are enriched in mutual inhibitory interactions and mutual growth promotions, while it is rare to find cases in which one strain promotes a second, but this second strain inhibits the first. This reciprocity is further enriched among strains derived from the same grain of soil, thus revealing spatial structuring of interactions.

These properties of the interaction network have emerged from a long evolutionary process, which we probed by juxtaposing interactions and phylogeny. We found that the interactions of an isolate can change dramatically even over short evolutionary time (indicated by very close 16S sequences), with evolution changing the production profiles more than the resistance profiles. Incorporating such fast evolution in a dynamic ecological model of antibiotic interactions, we find that most of the observed properties of the network are reproduced under a broad range of parameters. The community compositions are not static—increase in production of an antibiotic promotes resistance, which promotes sensitivity, and invites production again. As the community undergoes cycles with respect to different antibiotics, different combinations of production and resistance become favorable, which makes it evolutionary unstable. In our model both ecological interactions and continuous turnover of interaction phenotypes are required to maintain functional diversity.

Our work has several important limitations. Perhaps the main one is that interactions are measured in the lab and actual interactions in the soil may be more complex or different. We were also limited to studying only the interactions among *Streptomyces* strains; interactions between *Streptomyces* and other microbes could be of major importance. Higher order interactions, such as synergy or antagonism between natural products, or induction of small molecule production by other small molecules, are not captured by the pairwise measurements. Many of these shortcomings are inherent to most current studies of microbial species interactions. However, the systematic and high-throughput nature of the current study allows us to ask questions at the statistical level, and might therefore be less prone to some of these difficulties. Furthermore, the high-throughput interaction platform developed here and the simulations offer a natural foundation for many subsequent studies of microbial communities, which will address some of the above concerns, potentially yielding important biological insights. For example, it is now possible to probe how the statistical properties of networks, such as the relative significance of positive and negative interactions, are affected by media composition and the presence of other small molecules. This enables investigations of the regulatory roles of and epistatic effects between small molecules. It would also be interesting to see whether the effects of a sender on a receiver will be modified if the sender is co-incubated with the receiver. Finally, the interaction platform can be used to follow the evolutionary and ecological dynamics of synthetic laboratory communities of interacting microbial strains.

The observed network properties do not seem to correspond to an ecologically stable state maintained by antibiotic interactions alone. Instead, the model and observations suggest that they are supported by a constant evolutionary change. The distribution of production and resistance in the community is poised so that simple changes in production capabilities of a strain can alter its interactions with many other strains potentially to a great ecological advantage. This evolutionarily unstable ecological state seems complemented by the modular nature of the secondary metabolite gene clusters, which enable such changes and, thus, lead to turnover of interaction phenotypes of different strains and species. This continuous turnover might in turn be important for the emergence and maintenance of the modularity and clustering of small molecule production and resistance genes and their recruitment to mobile genetic elements [21]. This reasoning suggests a unified view of network structure, network evolution, and modularity of secondary metabolism to be further explored.

## Materials and Methods

### Sampling Grains, Isolation, and Spore Stock Preparation

We sampled four soil grains of soil by touching the soil with a dry needle tip, and lifting particles of less than 1 mg of wet weight. Three of the grains were 1 cm away from each other in one soil core, and the fourth grain was 10 cm away from a second soil core. The depth was approximately 2 cm below the surface. The sampling was performed in December from foliage-covered soil away from visible roots. Each grain was dried for 2 d, then suspended in dH_2_O, vortexed, sonicated, diluted, and plated on Streptomyces Isolation Media [7]. Plates containing five colonies or fewer were sampled in order to minimize potentially biasing interactions between emerging colonies. Isolates that exhibited the characteristic aerial mycelium pattern of *Streptomyces* were selected at random after 2 wk, and their genus identity later verified by sequencing. Five of the isolates were classified as genus *Kitasatospora* within the family *Streptomycetaceae* by the Ribosome Database Project [22]. Each isolate was restreaked once, then grown in TSB for 3 d, and 300 µl/plate was spread on four petri dishes containing Bennett's agar [7]. Plates were incubated for 14 d at 28°C. Spore lawns were harvested in 12 ml of 0.01% Tween 80, vortexed for 2 min, and filtered through 5 µm syringe filter to separate the spores from mycelium. The filtrate was centrifuged at 1,000 g for 10 min, and the spore pellet was resuspended in 1.1 ml of 20% glycerol, aliquoted, and frozen at −80°C. Each spore stock that we used was thawed only once. During stock preparation, tubes were kept on ice.

### Soil Properties

Bulk soil was sent to the Soil and Plant Tissue Testing Lab at the University of Massachusetts at Amherst. The soil pH is 5.5. The texture is loam with 46.7% sand, 42.1% silt, and 11.2% clay. Organic matter, 12.6%. NO_3_-N, 0 ppm. Mineral content: P, 7 ppm; K, 230 ppm; Ca, 1,511 ppm; Mg, 157 ppm. Micronutrients: B, 0.3 ppm; Mn, 7.1 ppm; Zn, 9.3 ppm; Cu, 0.3 ppm; Fe, 32.4 ppm; S, 28.8 ppm. Cation Exch Cap, 21.7 Meq/100 g.

### Interaction Media

Media for interactions: 15 g purified agar in 1 L d H_2_O, 2 g potato starch, 0.8 g casein, 1 g KNO_3_, 0.4 g K_2_HPO_4_, 0.2 g MgSO_4_, 30 mg CaCl_2_·2H_2_O, pH 7.2. All components were autoclaved separately in concentrated form, and all agar plates were made from the same autoclaved stocks. Resupply media was 18× concentrated interaction media with the exception of KHPO_4_, which was 36× concentrated, pH 7.0.

### Interaction Protocol

Black 96-well agar plates were robotically over-filled with agar, and before solidification a glass plate was lowered to 1.5 mm above the plate to flatten the agar meniscus. The glass plate was slid sideways upon solidification of the agar. The resulting agar columns were flat on top (to ensure good filter contact and high image quality), protruded above the edge of the plate (to ensure good contact with filter during conditioning), and well separated from neighboring wells (to prevent cross-talk). Since high pipetting accuracy was required, the aspirated amount was automatically adjusted based on the instantaneous agar temperature (∼50°C), care was taken to dip the pipette tips to the same depth in the agar reservoir, and room ventilation was turned off to prevent asymmetric cooling of the agar in the tips. Rectangular filters—polycarbonate 0.03 µm pore size—were placed over the agar plates. Each well was inoculated with 8 µl of spore stock. Due to the hydrophobicity of the filters, droplets above neighboring wells were well separated.

After 8 d of incubation, growth on each filter was imaged, and the filter removed. Filter images were used to discard data from defectively conditioned or contaminated wells. Plates were resupplied with a 20 µl droplet of resupply media, and dried in a fume hood for 90 min. Each plate was pinned from a source 96-well plate containing 100 µl/well of spore stock (∼10^7^ spores/ml). Source plates were kept between 4 and 8°C during pinning. Pins were sterilized between plates to prevent contamination of the source plate due to accidental contamination of the agar plates. The time of pinning of each plate was recorded, and it was placed upside down on a flatbed scanner so that the agar surface is 2 mm away from the scanner glass surface. The focusing plane of the scanners was correspondingly adjusted. To minimize agar drying, plates were sealed to the scanners with packing tape. Colonies were scanned approximately every 4 h. Temperature was maintained at 28°C, but jumped temporarily by 1°C after each scan. Plates were scanned for at least 15 d.

### Image Analysis

The appearance time for each colony (the first time point at which a colony becomes visible on the images) was manually determined using custom interactive software. Colonies associated with agar defects or contaminations were discarded. Aerial mycelium is apparent on the images as a fuzzy texture on top of the colonies (Figure 1D). Aerial mycelium inhibition was scored if there was no or very little (in comparison to non-conditioned) aerial mycelium coverage of the colony after 15 d.

### Interaction Frequencies Details

Twenty-five percent of the interactions are complete inhibitions, i.e. no visible growth of receiver colonies. One isolate inhibits itself. An additional 10% of interactions are partial inhibitions with colonies appearing at least 1 d later on conditioned media (for a total of 35%). The fraction of inhibitory interactions is 45%, if inhibitions of aerial mycelium formation are included.

### 16S rRNA Sequencing

Colonies were grown in TSB for 3 d, centrifuged at 1,000 g for 10 min, and resuspended in dH_2_0 three times. Cells were then resuspended in lyses buffed (PrepMan) and heated to 100°C for 10 min, centrifuged, and the supernatant was frozen at −20°C. 3 µl of this supernatant was added to 60 µl PCR mix containing 12 µl Qiagen Q-solution, 2.4 µl of 10 µM forward primer GAG AGT TTG ATC CTG GCT CAG, and reverse primer CGG CTA CCT TGT TAC GAC TTC. Samples were PCR amplified (95°C for 3 min, 35 cycles of 95°C for 1 min, 55°C for 1 min, 72°C for 1:30 min, and final extension at 72 deg for 7 min), and PCR products were sent for sequencing upon confirmation of existence of a product of the expected size (∼1.5 kb). Sequences from the forward and reverse primers had a significant overlap. Sequences are available through Genbank, accession numbers: JN020489–JN020551. The grain and isolate number within a grain is specified in the description for each sequence; e.g. G4_6 is the sixth isolate from grain four.

### Phenotypic Diversity of Isolates

We considered the profiles of two isolates distinct if they differed by more than 2 d in appearance time (the first time point in which a colony becomes visible on the images) for both replicates in at least three sender or three receiver positions. According to this measure, there are 42 distinct phenotypic profiles.

### Interaction Density and Sender-Receiver Asymmetry

For a *N*×*N* binary interaction matrix 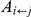 (one indicates an interaction, and zero no interaction), the frequency of interactions is 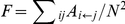, and the sender-receiver asymmetry is defined as 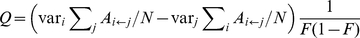. Matrices with negative *Q* are sender-determined, and with positive *Q* are receiver-determined. We obtain negative *Q* independently of how we threshold the inhibitory interactions and of whether or not we include aerial mycelium inhibitions.

### Calculating Phenotypic Distance Between a Pair of Sender/Receiver Profiles

The fraction of differences between profiles was calculated (after discarding defective and inconsistent replicas). The profiles were taken from a binary interaction matrix in which inhibitions were defined as delays in colony appearance time of more than 1 d. Increasing the threshold to 3 d (i.e. strong inhibitions) did not change the qualitative findings of Figure 3. However, inclusion of aerial mycelium inhibitions renders the statistics of Figure 3D insignificant.

### Calculating Phylogenetic Distance

Sequences were aligned to a universal 16S rRNA template using the Ribsomal Database Project website [22]. For each pair of sequences, only positions for which both sequences have high-quality values from the sequencing trace were considered; the rest were treated as missing values. Phylogenetic distance was computed as the fraction of differences (in high-quality positions). Alignment gaps were counted as normal differences.

### Network Motifs

For the measured network of positive and negative interactions (without weights), we generated an ensemble of random networks that have the same number of ingoing and outgoing arrows of positive and negative interactions for each isolate. Networks were randomized by taking random pairs of single arrows (between different isolates) and swapping the isolates on which they end, provided the two arrows created by the swap do not exist already or correspond to missing or defective experimental values. (In this way, the missing or defective values of the matrix were kept in place.) Each cycle consisted of swapping one pair of positive and one pair of negative arrows. This operation was performed thousands of times before selecting each random ensemble representative. For each random network the frequency of each of the six pairwise motifs was calculated, without counting any of the diagonal matrix elements. The motif significance (*p* value) was calculated as the fraction of random networks that have more extreme motif frequency than that for the observed network. The protocol was analogous for the matrices resulting from the eco-evolutionary model, which had only negative interactions and no missing or defective values.

### Eco-Evolutionary Model

*Ecology:* Each strain *i* is characterized by an array *Z_iα_*, specifying whether it is producer (*P*), resistant (*R*), or sensitive (*S*) to antibiotic *α*. Let *A_i←j_* be the binary matrix of inhibitory interactions. Strain *j* inhibits strain *i*, i.e. *A_i←j_* = 1 , if *Z_jα_ = P* and *Z_iα_* = *S* for any *α*. Let *n_i_* be the fractional abundances of different strains (summing to one). The “fitness” of *i* is 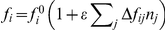, where 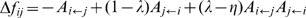. At each time step *N* individuals are drawn from different species with relative probafobilities 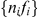. *λ* and *η* are (positive) ecological parameters controlling the direct benefit of inhibiting neighbors and the consequence of mutual inhibition (the level of protection by inhibition is 1−*η*), and 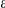 is the intensity of selection within an ecological cycle (which we specify through 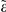 by 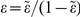). *λ* = 0 means that inhibition is a zero-sum game; in the other extreme *λ* = 1 means complete spite (no direct benefit for the inhibitor). Production or resistance of an antibiotic incurs a multiplicative fitness cost, so that 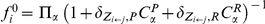 is the bare fitness reflecting the costs 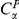 and 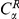 of production and resistance (antibiotic dependent), and *δ* is the Kronecker delta. *Evolution*: each antibiotic position in each of the *N* individuals mutates within the SRP space of possibilities with probability specified by a set of transition rates: 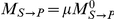, 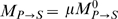, 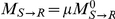, 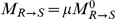, 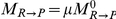, 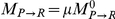.

### Parameters for Main Text Figures

*N* = 10^6^, 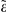 = 0.05, *λ* = 0.15, *η* = 0.7, 40 antibiotics. 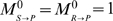, 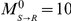, 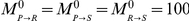, and 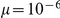. The relative mutation rates assume that loss of function is more likely than gain of function, and gain of resistance is easier than gain of production. The no interaction case in Figure 4E corresponds to 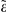 = 0. Figures S2 and S3 explore the behavior of the model for other parameters.

### Model Extensions

We examined the behavior of the model when different antibiotics have different production costs rather than identical costs. The production costs were uniformly distributed in the interval ranging from the resistance cost up to the maximal cost for which a producer can invade a sensitive strain. We discovered that this extends the region over which we observed balanced interaction matrices, and leads to receiver-determined matrices at large resistance costs (Figure S7A). We also added an evolutionary operator that mimics more closely within-population HGT—rates of change towards production and resistance of an antibiotic are proportional to the abundance of production and resistance to that antibiotic in the population (rather than being constants). With probability of 10^−4^ an organism pairs with another random organism and gains a production or resistance for an arbitrary antibiotic of the donor. Adding within-population HGT (while keeping the mutations) did not qualitatively change the results (Figure S7B).

## Supporting Information

Figure S1 Sender and receiver degrees are negatively correlated. (A) Each point shows the sender and receiver degrees for an isolate. Least square linear fit is also shown. Here, inhibition is defined as a 1 d delay in appearance time or sporulation inhibition. (B) Sender degree is not correlated with growth on non-conditioned media expressed as colony appearance time in days.
(PDF)

Figure S2 When inhibiting others severely reduces their ability to inhibit back (small η), a qualitatively new behavior emerges at high resistance costs. There is a sharp transition to very dense interaction matrices (every species inhibits almost every other) as the resistance cost is increased (black region). The balancing feedback loop fails because antibiotic production can provide more effective defense than the costly resistance. The system then falls into a state maintained by mutual inhibition, where many antibiotics are being produced, resistance to any individual antibiotic therefore confers no benefit, and the better defense mechanism becomes the production of yet more antibiotics. The model still generates balanced and sender-determined interaction matrices, but only in a particular region of the parameter space corresponding to low resistance costs and high production costs (green shades). Same simulation parameters as Panel 4B but with η = 0.05.
(PDF)

Figure S3 Sensitivity analysis of simulation outcomes. Presented are the density of interactions and the sender-receiver asymmetry (colored according to the legend introduced in Figure 4C) for different parameters. In each panel we specify how the model parameters differ than those in Figure 4D. A mutation rate of *μ* = 5 10^−7^ was used unless indicated otherwise. Simulations were performed for values denoted by blue dots, and nearest neighbor interpolation was used. For each point the results are averages over 8 randomly drawn matrices of 64 isolates, and 10 time points separated by 1,000 generations. Blue regions indicate missing simulations. (A) The critical η below which a high interaction density solutions appear is not strongly dependent on λ. We set the production cost to half the maximum cost for which antibiotic producers can invade a sensitive population, and explored the resulting matrices for different η and resistance costs at two values of λ. From these diagrams we can read out the maximum η, η^*^, that leads to high interaction density solutions. In both cases the critical value is around 0.15. (B) The statistical properties of interactions are not sensitive to λ. (C) The statistical properties of interactions depend only weakly on the population size and the evolutionary rate expressed as number of mutation events per population per generation. These simulations were performed with 20 antibiotics rather than 40. (D) Different relative mutation rates lead to qualitatively similar behavior. Left panel: Different probabilities to lose or gain function. Right panel: all mutation rates are identical. (E) Matrices that result with strong interactions: ε = 0.96 (close to the maximum of 1) for η = 0.7 (left panel) and for η = 0.05 (right panel). The qualitative behavior is the same as in Figures 4D and S2, but the fraction of interactions is lower, i.e. less balanced. Notice that the costs of production and resistance are larger than those in Figure 4D because the interaction strength sets a scale for the costs.
(PDF)

Figure S4 Dynamic properties of evolved model communities. (A) An interaction matrix resulting from randomly drawing a hundred isolates from an in silico community, and sorting them by phylogeny. (B) Number of generations since separation (*x*-axis) versus the sender (green) and receiver (magenta) profile distances (*y*-axis) for all isolate pairs. (C) Closely related isolates are less likely to inhibit each other. Plotted is the probability of inhibition for all pairs of “isolates” phylogenetically separated by less than a given number of generations. (D) The steady state is maintained through turnover of production and resistance profiles. Shown is the fraction of surviving combinations of production and resistance between two time points as a function of the number of generations. Same parameters and solution as in Figure 4C are used.
(PDF)

Figure S5Functional diversity is maintained through interplay between ecology and evolution. (A) Diversity as a function of evolution rate for simulations with interactions (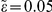, black circles) and without interactions (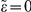, red dots). Two organisms are considered functionally distinct if they interact differently (as senders or receivers) with an existing organism. The ecosystem is ecologically unstable—there is no diversity at low evolutionary rates. However, in the presence of interactions, diversity increases rapidly as a function of the rate of evolutionary events in the population. Diversity is expressed as the exponential of the Shannon-Wiener Index, and the evolutionary rate per generation per population is expressed as µ*N*. *λ* = 0.15, *η* = 0.05, 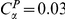, 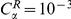, 20 antibiotics, *N* = 531,440. (B) The ratio of diversity with interactions turned on to diversity with interactions turned off increases as a function of the evolutionary rate per population per population. The increase is approximately linear (blue dotted line has a slope of one). Data for different evolutionary rates and population sizes is presented. Parameters, as in (A) and *N* = 19,680 (dot), *N* = 59,048 (x), *N* = 177,144 (+), *N* = 531,440 (circle), *N* = 1,594,320 (triangle), *N* = 4,782,968 (diamond).(PDF)

Figure S6 Continuous analysis of intra- versus inter-grain statistics of interactions reveals enrichment for reciprocity. (A) The cumulative distribution of appearance times for all pairs (black) and pairs from the same grain (green) are almost identical. (B). The cumulative distribution for the difference of appearance times for pairs of isolates on their reciprocal conditioned media. The distribution for all pairs is given in black and for pairs from the same grain in green. Apparent is enrichment for reciprocal/symmetric interactions. Blue line indicates the position of maximal difference between distributions. (C) We binary classify the pairs as reciprocal or non-reciprocal (using the maximal difference found in B), and compare the intra-grain frequency of reciprocal pairs for the actual grains (green) and randomized grains (black histogram). Aerial mycelium inhibitions are treated as complete inhibitions and set to appearance time of 10 d.
(PDF)

Figure S7 Model extensions. (A) Different antibiotics have different production costs. Top strip: statistical properties for simulations with antibiotic production costs uniformly distributed in the interval from the resistance cost up to the maximal cost for which a producer can benefit from inhibiting a sensitive strain. Different squares correspond to different resistance costs. Main: all antibiotics have the same production and resistance costs. This leads to mutual inhibition when inhibition is an efficient defense mechanism (identical to Figure S2). We realized that states of mutual inhibition are, at least to some extent, an artifact of having antibiotics with identical production and resistance costs. In the more biologically realistic scenario in which antibiotics have diverse production costs, the degeneracy between the antibiotics will be broken. Less costly antibiotics eventually will become dominant, therefore selecting for their resistance and leading the system to lower interaction density. This made us expect that broadly distributed production costs would extend the region of balanced interaction frequency at high resistance costs. Indeed, by simply utilizing an array of antibiotics with broadly distributed production costs we automatically obtained communities that are diverse and of balanced interaction frequency, almost independently of the resistance cost (top). With distributed production costs, the matrix properties change gradually from sender-determined to receiver-determined as we increase the cost of resistance. (B) A different evolutionary scheme leads to qualitatively similar results. In the mutational scheme used throughout the article the probability to gain and lose functions are constants independent of the composition of the population. To these mutations, we add a probability to gain function (production or resistance) that is proportional to abundance of the function in the population. In this way we better mimic the effect of horizontal gene transfer within the population. With probability of 10^−4^ an organism pairs with another random organism and gains a production or resistance for an arbitrary antibiotic of the donor.
(PDF)

Figure S8 Time-lapse images of isolates growing on media conditioned by other isolates. Sample only; the full dataset can be found online at http://kishony.med.harvard.edu/Vetsigian_sup_movie_strips/. Presented is the growth of one isolate on eight different conditioned media. Each interaction is labeled by “r∶x, s∶y,” where x and y are the identifying numbers for the receiver and the sender, and is present in two replicates. Images are shown for a subset of the measured time points. Above each image is specified the time after inoculation of the receiver, expressed in days. Red arrows indicate the colony appearance time; they point between images if colonies appeared at one of the omitted images. Red “S” specifies instances of scored sporulation/aerial mycelium inhibition.
(PDF)
