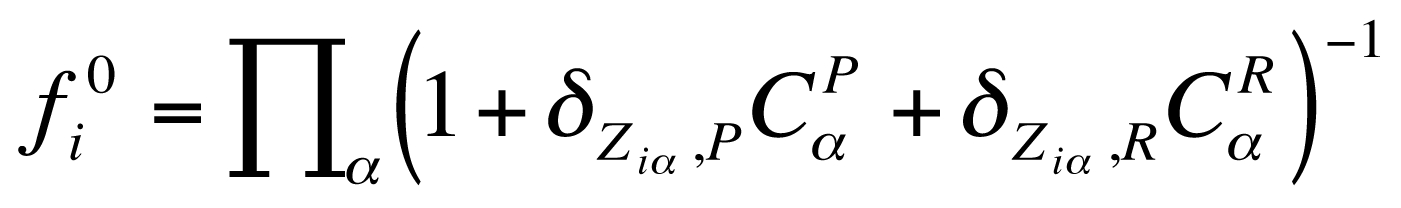# Correction: Structure and Evolution of *Streptomyces* Interaction Networks in Soil and In Silico

**DOI:** 10.1371/annotation/1d584443-c6b8-423b-8027-5f9034d4599f

**Published:** 2011-12-03

**Authors:** Kalin Vetsigian, Rishi Jajoo, Roy Kishony

In the Materials and Methods section, there was an error in the equation of the penultimate sentence in the section "Eco-Evolutionary Model." The indices of Z should be i,alpha and the Z should be curly as on the previous page. Please view the correct equation here: