# Supplementary figures and images for: Structure and Evolution of Streptomyces Interaction Networks in Soil and In Silico
Source: PLoS Biol. 2011 Oct 25;9(10):e1001184. doi: 10.1371/journal.pbio.1001184 (PMC3201933; doi:10.1371/journal.pbio.1001184)

**A.**

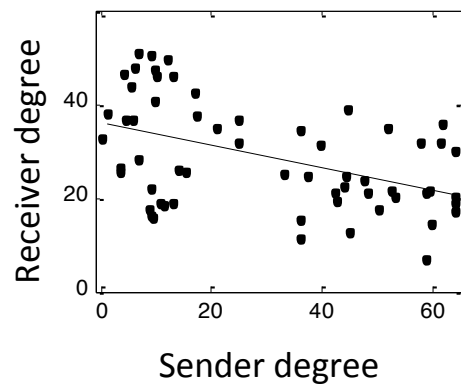

**B.**

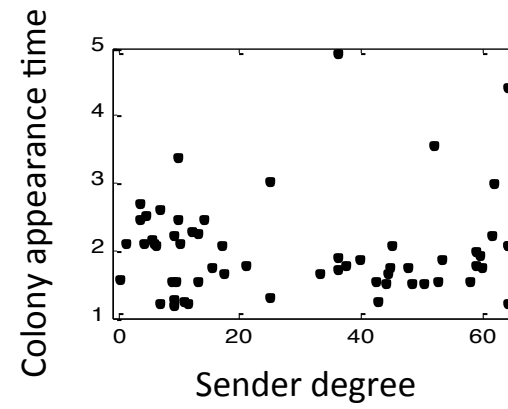

Supplement: Figure S1 — Sender and receiver degrees are negatively correlated. (A) Each point shows the sender and receiver degrees for an isolate. Least square linear fit is also shown. Here, inhibition is defined as a 1 d delay in appearance time or sporulation inhibition. (B) Sender degree is not correlated with growth on non-conditioned media expressed as colony appearance time in days. (PDF) [file pbio.1001184.s001.pdf]

$$\eta = 0.05$$

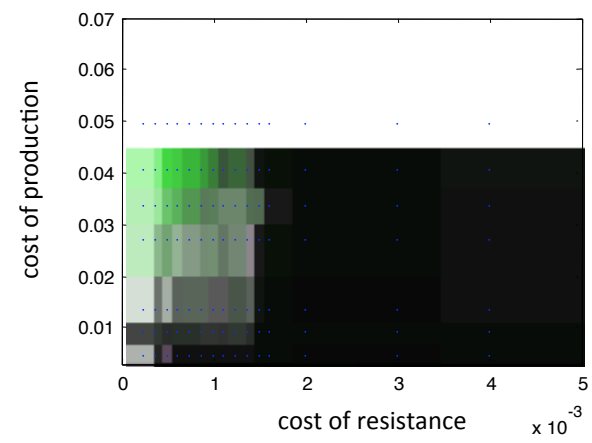

Supplement: Figure S2 — When inhibiting others severely reduces their ability to inhibit back (small η), a qualitatively new behavior emerges at high resistance costs. There is a sharp transition to very dense interaction matrices (every species inhibits almost every other) as the resistance cost is increased (black region). The balancing feedback loop fails because antibiotic production can provide more effective defense than the costly resistance. The system then falls into a state maintained by mutual inhibition, where many antibiotics are being produced, resistance to any individual antibiotic therefore confers no benefit, and the better defense mechanism becomes the production of yet more antibiotics. The model still generates balanced and sender-determined interaction matrices, but only in a particular region of the parameter space corresponding to low resistance costs and high production costs (green shades). Same simulation parameters as Panel 4B but with η = 0.05. (PDF) [file pbio.1001184.s002.pdf]

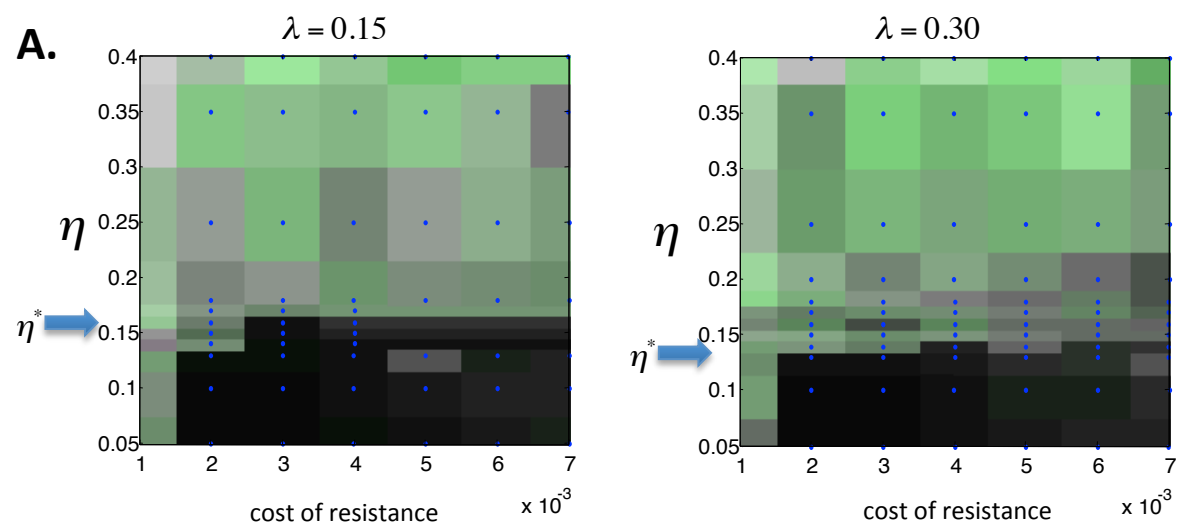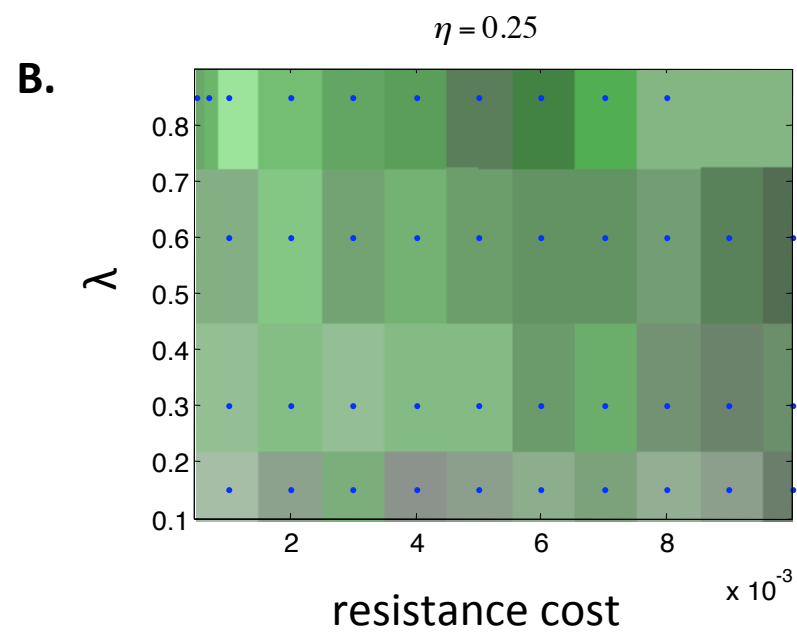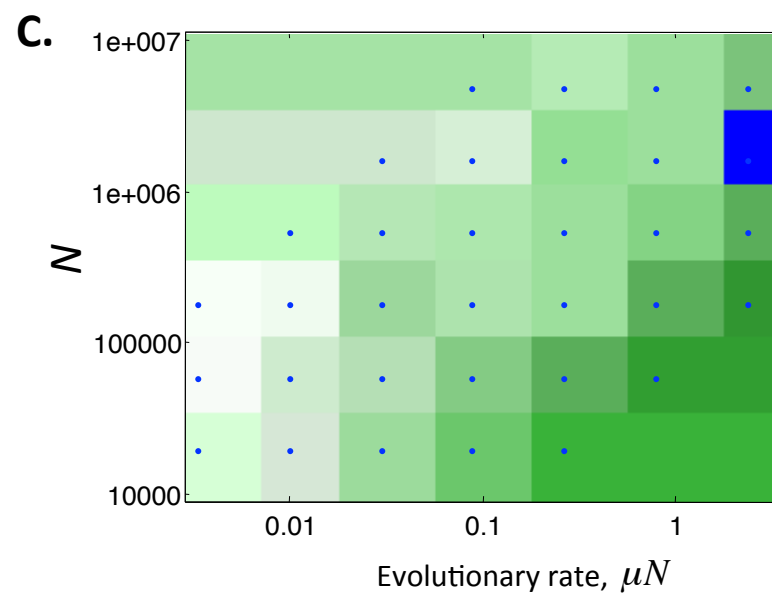

**D.**

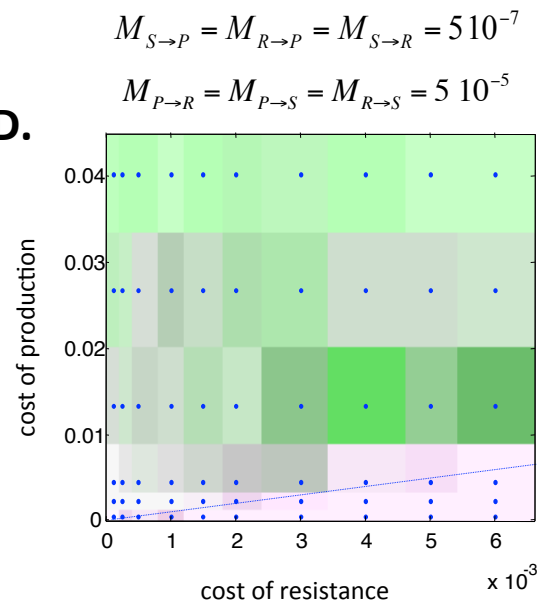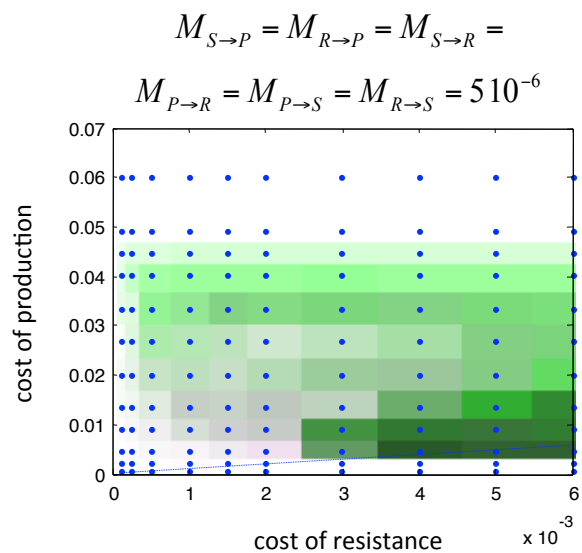

**E.**

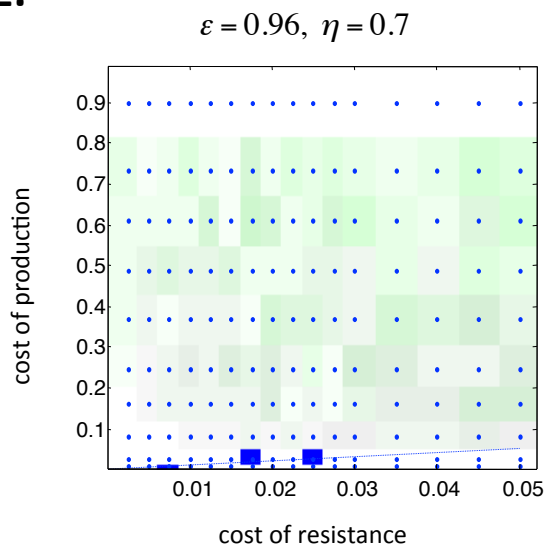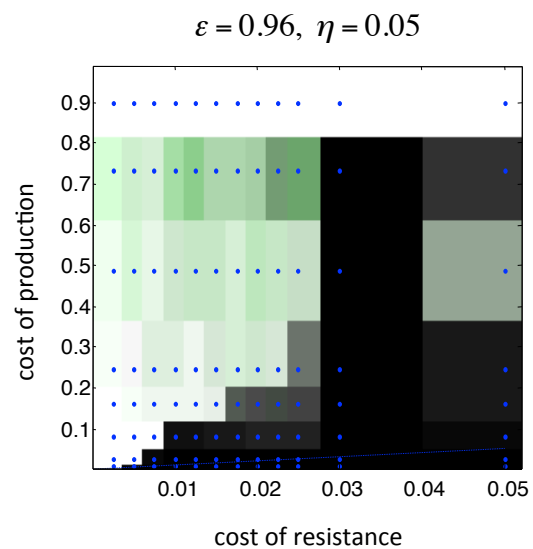

Supplement: Figure S3 — Sensitivity analysis of simulation outcomes. Presented are the density of interactions and the sender-receiver asymmetry (colored according to the legend introduced in Figure 4C) for different parameters. In each panel we specify how the model parameters differ than those in Figure 4D. A mutation rate of μ = 5 10−7 was used unless indicated otherwise. Simulations were performed for values denoted by blue dots, and nearest neighbor interpolation was used. For each point the results are averages over 8 randomly drawn matrices of 64 isolates, and 10 time points separated by 1,000 generations. Blue regions indicate missing simulations. (A) The critical η below which a high interaction density solutions appear is not strongly dependent on λ. We set the production cost to half the maximum cost for which antibiotic producers can invade a sensitive population, and explored the resulting matrices for different η and resistance costs at two values of λ. From these diagrams we can read out the maximum η, η*, that leads to high interaction density solutions. In both cases the critical value is around 0.15. (B) The statistical properties of interactions are not sensitive to λ. (C) The statistical properties of interactions depend only weakly on the population size and the evolutionary rate expressed as number of mutation events per population per generation. These simulations were performed with 20 antibiotics rather than 40. (D) Different relative mutation rates lead to qualitatively similar behavior. Left panel: Different probabilities to lose or gain function. Right panel: all mutation rates are identical. (E) Matrices that result with strong interactions: ε = 0.96 (close to the maximum of 1) for η = 0.7 (left panel) and for η = 0.05 (right panel). The qualitative behavior is the same as in Figures 4D and S2, but the fraction of interactions is lower, i.e. less balanced. Notice that the costs of production and resistance are larger than those in Figure 4D because the interact [file pbio.1001184.s003.pdf]

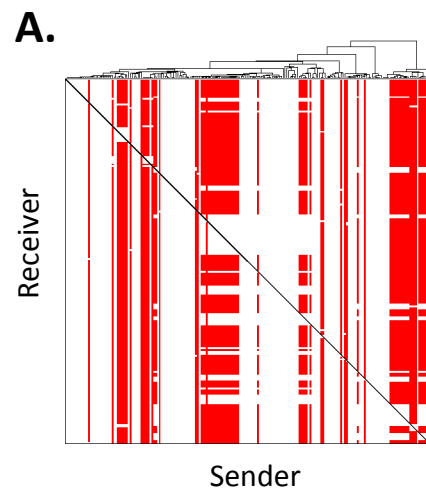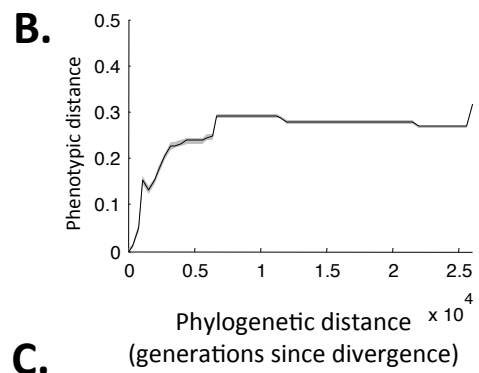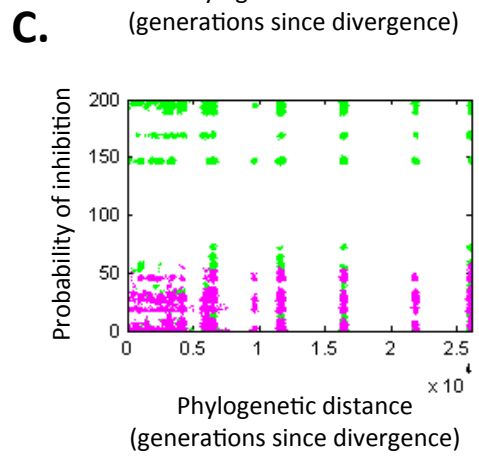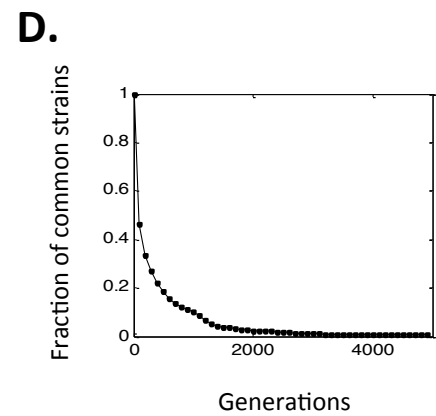

Supplement: Figure S4 — Dynamic properties of evolved model communities. (A) An interaction matrix resulting from randomly drawing a hundred isolates from an in silico community, and sorting them by phylogeny. (B) Number of generations since separation (x-axis) versus the sender (green) and receiver (magenta) profile distances (y-axis) for all isolate pairs. (C) Closely related isolates are less likely to inhibit each other. Plotted is the probability of inhibition for all pairs of “isolates” phylogenetically separated by less than a given number of generations. (D) The steady state is maintained through turnover of production and resistance profiles. Shown is the fraction of surviving combinations of production and resistance between two time points as a function of the number of generations. Same parameters and solution as in Figure 4C are used. (PDF) [file pbio.1001184.s004.pdf]

**A.**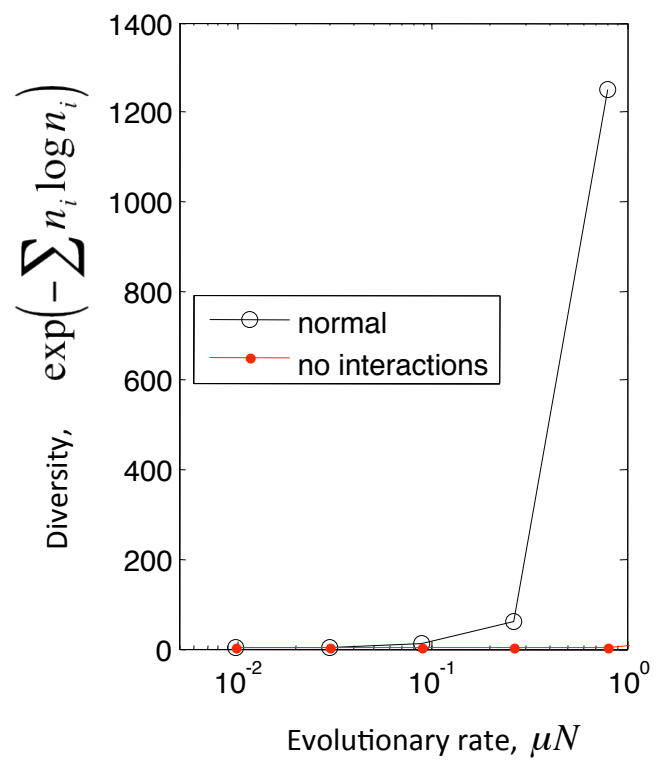**B.**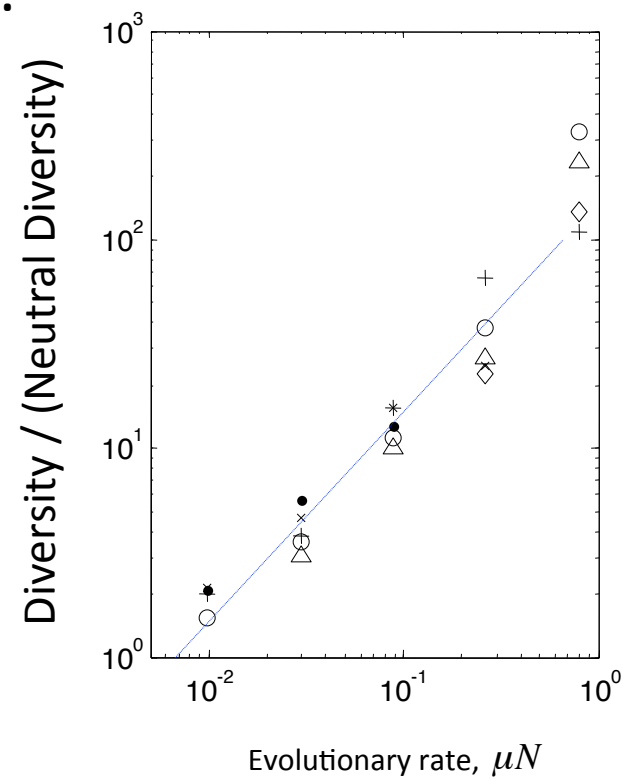

Supplement: Figure S5 — Functional diversity is maintained through interplay between ecology and evolution. (A) Diversity as a function of evolution rate for simulations with interactions (, black circles) and without interactions (, red dots). Two organisms are considered functionally distinct if they interact differently (as senders or receivers) with an existing organism. The ecosystem is ecologically unstable—there is no diversity at low evolutionary rates. However, in the presence of interactions, diversity increases rapidly as a function of the rate of evolutionary events in the population. Diversity is expressed as the exponential of the Shannon-Wiener Index, and the evolutionary rate per generation per population is expressed as µN. λ = 0.15, η = 0.05, , , 20 antibiotics, N = 531,440. (B) The ratio of diversity with interactions turned on to diversity with interactions turned off increases as a function of the evolutionary rate per population per population. The increase is approximately linear (blue dotted line has a slope of one). Data for different evolutionary rates and population sizes is presented. Parameters, as in (A) and N = 19,680 (dot), N = 59,048 (x), N = 177,144 (+), N = 531,440 (circle), N = 1,594,320 (triangle), N = 4,782,968 (diamond). (PDF) [file pbio.1001184.s005.pdf]

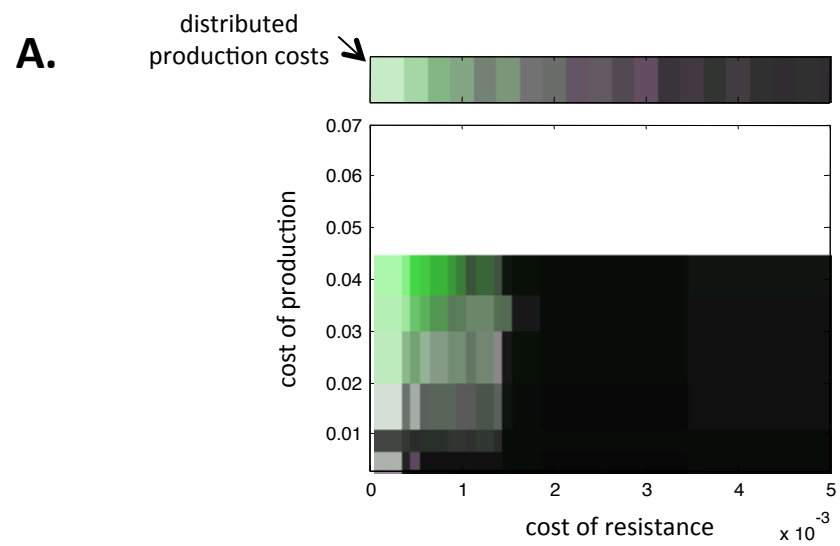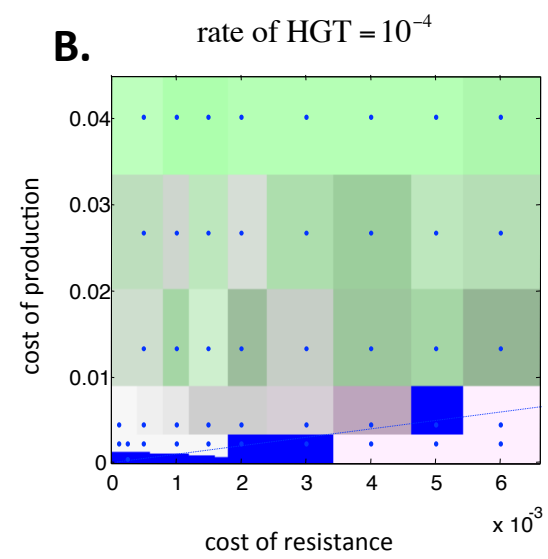

Supplement: Figure S7 — Model extensions. (A) Different antibiotics have different production costs. Top strip: statistical properties for simulations with antibiotic production costs uniformly distributed in the interval from the resistance cost up to the maximal cost for which a producer can benefit from inhibiting a sensitive strain. Different squares correspond to different resistance costs. Main: all antibiotics have the same production and resistance costs. This leads to mutual inhibition when inhibition is an efficient defense mechanism (identical to Figure S2). We realized that states of mutual inhibition are, at least to some extent, an artifact of having antibiotics with identical production and resistance costs. In the more biologically realistic scenario in which antibiotics have diverse production costs, the degeneracy between the antibiotics will be broken. Less costly antibiotics eventually will become dominant, therefore selecting for their resistance and leading the system to lower interaction density. This made us expect that broadly distributed production costs would extend the region of balanced interaction frequency at high resistance costs. Indeed, by simply utilizing an array of antibiotics with broadly distributed production costs we automatically obtained communities that are diverse and of balanced interaction frequency, almost independently of the resistance cost (top). With distributed production costs, the matrix properties change gradually from sender-determined to receiver-determined as we increase the cost of resistance. (B) A different evolutionary scheme leads to qualitatively similar results. In the mutational scheme used throughout the article the probability to gain and lose functions are constants independent of the composition of the population. To these mutations, we add a probability to gain function (production or resistance) that is proportional to abundance of the function in the population. In this way we better mimic the effect of horizontal ge [file pbio.1001184.s007.pdf]
