# Supplementary material for: Structure and Evolution of Streptomyces Interaction Networks in Soil and In Silico
Source: PLoS Biol. 2011 Oct 25;9(10):e1001184. doi: 10.1371/journal.pbio.1001184 (PMC3201933; doi:10.1371/journal.pbio.1001184)
Supplement: Figure S6 — Continuous analysis of intra- versus inter-grain statistics of interactions reveals enrichment for reciprocity. (A) The cumulative distribution of appearance times for all pairs (black) and pairs from the same grain (green) are almost identical. (B). The cumulative distribution for the difference of appearance times for pairs of isolates on their reciprocal conditioned media. The distribution for all pairs is given in black and for pairs from the same grain in green. Apparent is enrichment for reciprocal/symmetric interactions. Blue line indicates the position of maximal difference between distributions. (C) We binary classify the pairs as reciprocal or non-reciprocal (using the maximal difference found in B), and compare the intra-grain frequency of reciprocal pairs for the actual grains (green) and randomized grains (black histogram). Aerial mycelium inhibitions are treated as complete inhibitions and set to appearance time of 10 d. (PDF) [file pbio.1001184.s006.pdf]

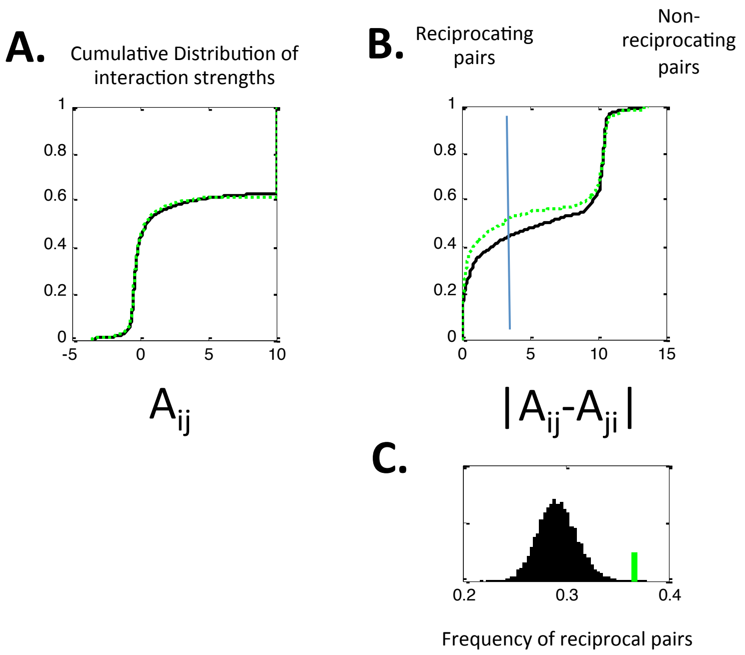
A.
Cumulative Distribution of interaction strengths
1
0.8
0.6
0.4
0.2
0
-5
0
5
10
Aij
B.
Reciprocating pairs
Non-reciprocating pairs
1
0.8
0.6
0.4
0.2
0
0
5
10
15
|Aij-Aji|
C.
0.2
0.3
0.4
Frequency of reciprocal pairs
