# Supplementary material for: Structure and Evolution of Streptomyces Interaction Networks in Soil and In Silico
Source: PLoS Biol. 2011 Oct 25;9(10):e1001184. doi: 10.1371/journal.pbio.1001184 (PMC3201933; doi:10.1371/journal.pbio.1001184)

Figure S8. Sample from an online database of isolates growing on media conditioned by other isolates. Complete dataset is at **http://kishony.med.harvard.edu/Vetsigian_sup_movie_strips/** .

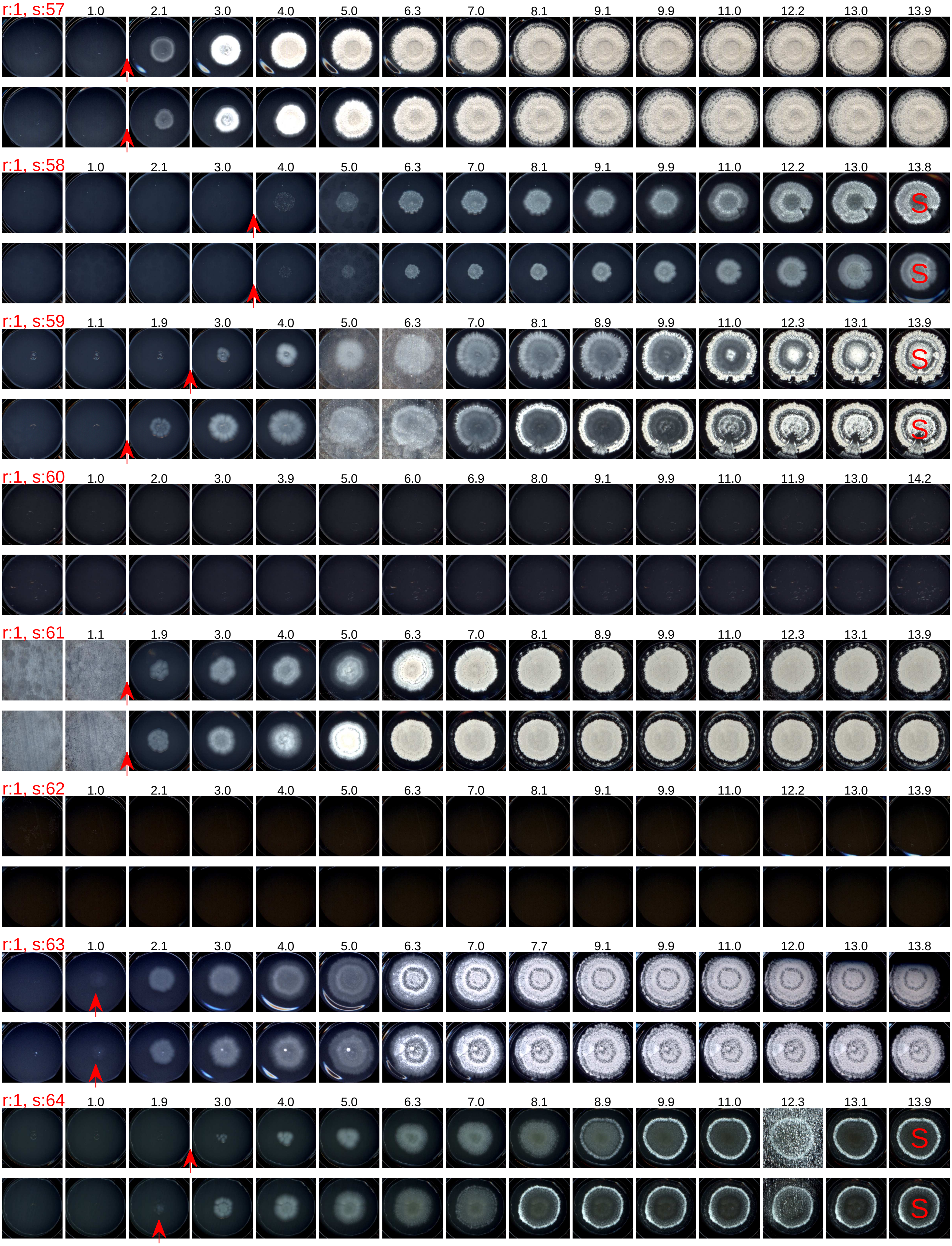

Supplement: Figure S8 — Time-lapse images of isolates growing on media conditioned by other isolates. Sample only; the full dataset can be found online at http://kishony.med.harvard.edu/Vetsigian_sup_movie_strips/. Presented is the growth of one isolate on eight different conditioned media. Each interaction is labeled by “r∶x, s∶y,” where x and y are the identifying numbers for the receiver and the sender, and is present in two replicates. Images are shown for a subset of the measured time points. Above each image is specified the time after inoculation of the receiver, expressed in days. Red arrows indicate the colony appearance time; they point between images if colonies appeared at one of the omitted images. Red “S” specifies instances of scored sporulation/aerial mycelium inhibition. (PDF) [file pbio.1001184.s008.pdf]
